# Comparing the Antibacterial and Functional Properties of Cameroonian and Manuka Honeys for Potential Wound Healing—Have We Come Full Cycle in Dealing with Antibiotic Resistance?

**DOI:** 10.3390/molecules200916068

**Published:** 2015-09-02

**Authors:** Joshua Boateng, Keshu Nso Diunase

**Affiliations:** Department of Pharmaceutical, Chemical & Environmental Sciences, Faculty of Engineering and Science, University of Greenwich, Medway, Central Ave. Chatham Maritime, Kent ME4 4TB, UK; E-Mail: duinase@yahoo.com

**Keywords:** antibacterial activity, antibacterial natural products, antibiotic resistance, anti-oxidation activity, Cameroonian honey, infection, Manuka honey, minimum inhibitory concentration, wound healing

## Abstract

The increased incidence of bacterial resistance to antibiotics has generated renewed interest in “traditional” antimicrobials, such as honey. This paper reports on a study comparing physico-chemical, antioxidant and antibacterial characteristics (that potentially contribute in part, to the functional wound healing activity) of Cameroonian honeys with those of Manuka honey. Agar well diffusion was used to generate zones of inhibition against *Escherichia coli*, *Pseudomonas aeruginosa* and *Staphylococcus aureus* while broth dilutions were used to study the minimum inhibitory concentrations (MICs). Non-peroxide activity was investigated by catalase for hydrogen peroxide reduction. The Cameroonian honeys demonstrated functional properties similar to Manuka honey, with strong correlations between the antioxidant activity and total phenol content of each honey. They were also as effective as Manuka honey in reducing bacteria load with an MIC of 10% *w*/*v* against all three bacteria and exhibited non-peroxide antimicrobial activity. These Cameroon honeys have potential therapeutic activity and may contain compounds with activity against Gram positive and Gram negative bacteria. Antibacterial agents from such natural sources present a potential affordable treatment of wound infections caused by antibiotic resistant bacteria, which are a leading cause of amputations and deaths in many African countries.

## 1. Introduction

A wound refers to an interruption in the defensive role of the skin in protecting against harmful environmental agents [1]. When injury occurs, the continuity of the epithelium is lost, which evokes wound healing, involving several phases (hemostasis, inflammation, proliferation, migration and maturation) [2]. During the proliferation stage, tissues may be prone to bacterial infection leading to dark granulation of the tissues which can delay wound healing. There has been a recent surge in interest for wound management products of natural origin including honey [3]. Tonks *et al.* [4] showed that a co-culture of MonoMac-6 cells (MM6; a monocytic cell line and precursor of macrophages) with various honeys for 2 h, caused a reduction in reactive oxygen intermediates whilst increasing the release of the potent pro-inflammatory cytokine, tumor necrosis factor-α. In a follow up study, the effect of Manuka, pasture and jelly bush honeys on the release of inflammatory cytokines from MM6 cells were investigated [5]. ELISA assays were used to test cell cultures for tumor necrosis factor-α (TNF-α) and interleukin (IL)-1β and IL-6. Their results showed that all three honeys significantly increased the TNF-α, IL-1β and IL-6 release from MM6 cells (and human monocytes) when compared with untreated and artificial-honey-treated cells (*p* < 0.001). The study concluded that the effect of honey on wound healing may in part be related to the stimulation of inflammatory cytokines from monocytic cells [5]. Ranzato *et al.* [6] investigated the wound healing properties of three different mono-floral honeys (acacia, buckwheat and Manuka) by their action on human fibroblasts with an *in vitro* scratch wound healing model. Their results showed enhanced wound healing action because of activity on fibroblasts, but to different extents and via different mechanisms of action, for the different honeys tested. Further, they observed that acacia and buckwheat, but not Manuka, induced significant increases in the release of interleukin-4 (IL-4), IL-6, and IL-8, indicating a correlation between interleukin up-regulation and wound closure efficiency [6].

Honey is produced from nectar collected by bees (*Apis mellifera*) with a density of about 1.36 g/mL (36% more dense than water) [7] and has been valued for its biomedical activity in treating wounds such as burns, diabetic, pressure and leg ulcers [4]. As far back as 2100–2000 BC, the first medicinal writings about the use of honey as a drug to treat ulcers, had been recorded by Sumeria [8]. In addition, Aristotle in 384–322 BC mentioned the use of honey for treating sore eyes and wounds. Honey is also reported to be effective against a broad range of wound types such as leg ulcers, burns, scratches, boils, amputation, burst abdominal wound, cracked nipples, fistulas, diabetic, malignant, leprosy, traumatic, cervical, varicose ulcers, septic and surgical wounds, as well as wounds of the abdominal wall and perineum [9].

Natural honey contains carbohydrates, water and other minor but important ingredients including proteins, minerals, phytochemicals and antioxidants. The minor ingredients account for its medical and biological properties for treating infections, burns, wounds and ulcers [10]. The major (about 82%) component of honey is carbohydrates and most of this is comprised of sugars. The majority of sugars in honey are fructose (38.2%) and glucose (31.2%), sucrose (0.7%–1%), disaccharides (approximately 9%) some trisaccharides and higher saccharides [11].

The two most common innovative commercial products currently available on the market are Manuka honey [12] and Surgi-honey [13]; and various studies on their biomedical properties have demonstrated their effectiveness in wound management. The medicinal benefits of honey have gained significant interest as more medical professionals and scientific researchers acknowledge its antibacterial efficiency [14]. This is due in part to factors such as increased bacterial resistance to first line broad spectrum antibiotics, the significant reduction in the number of new antibiotics approved for market and the various complications involved with chronic wounds (e.g., leg and diabetic ulcers) which can lead to amputations and sometimes death [15]. Molan reviewed the clinical evidence from various randomized control trials (RCTs) conducted between 2006 and 2011, for the effectiveness of honey based dressings in healing of wounds [16]. The review revealed that the use of honey and honey based dressings had increased from 1965 in the year 2006 to 3656 by the year 2011. It was further observed that there was an increase in the range of honey types used as well as the types of wounds that were treated [16]. The review further noted that the above variables (different honeys, wound types and dressings) present challenges which make it difficult for Clinicians to make informed decisions about the clinical efficacy of honey in a systematic manner. It was concluded that proper characterization of the bioactivities of different honey types was required for a systematic and reliable comparison of their potential wound healing performance [16].

Although the commercially available Manuka and Surgi honeys have been established as the medical honeys for wound care in Australia and Europe respectively [17], very few studies highlight the use of traditional African honeys for potential wound care [18,19,20]. This is important, given that most African natives who cannot afford appropriate medical care use their locally produced honeys for various therapeutic purposes without knowing the scientific mechanism behind their activity. Furthermore, diabetic chronic wounds, injuries caused by activities such as farming and surgical wounds, present significant challenges to African Clinicians in controlling associated infections [20]. No study has been undertaken to test the antimicrobial efficacy of local Cameroonian honeys against bacteria commonly residing in chronic wounds. This study therefore aims to systematically investigate the physico-chemical, anti-oxidant and antibacterial (*in vitro*) characteristics of two types of Cameroonian honeys and compare with those of medical grade Manuka honey.

## 2. Results

### 2.1. Physico-Chemical Properties

Table 1 summarizes the physico-chemical properties of the three honey samples tested. The mean densities (g/mL) for the honeys ranged from 1.47 ± 0.04 for M, 1.62 ± 0.01 for CS and 1.66 ± 0.02 for CW and the differences were significant (*p* < 0.05). The local Cameroonian honeys were more acidic with a mean pH value of 4.18 ± 0.01 (CS) and 4.1 ± 0.08 (CW) compared to 4.3 ± 0.04 for M, though the differences were not significant (*p* = 0.05). There was no significant difference in the mean sugar contents per 100 g of honey with values of 82% ± 0.58% (M), 79.7% ± 0.58% (CS) and 76.8% ± 0.15% (CW).

**Table 1:** Physicochemical parameters of honey samples (average ± standard deviation, *n* = 3).

Parameters	Manuka (M)	Cameroon Standard (CS)	Cameroon Wild (CW)
Density (g/mL)	1.47 ± 0.04 _c_	1.62 ± 0.01 _b_	1.66 ± 0.02 _a_
Moisture %/100 g	17.40 ± 0.00 _b_	17.80 ± 0.12 _a_	20.40 ± 0.40 _a_
pH	4.30 ± 0.04 _a_	4.18 ± 0.01 _a_	4.10 ± 0.08 _a_
Sugar Content %/100 g	82.00 ± 0.58 _a_	79.70 ± 0.58 _a_	76.80 ± 0.15 _a_
Total phenol (mg GAEs/kg)	103.99 ± 1.68 _a_	86.29 ± 9.87 _a_	73.18 ± 8.11 _b_
FRAP values (Fe^2+^ µM/kg)	988.60 ± 0.34 _c_	1242.20 ± 0.59 _b_	1284.50 ± 0.28 _a_

The subscripts (_a_, _b_, and _c_) represent which honeys are significantly different by the post hoc *t*-test, *p* = 0.05. Where a given parameter for particular honey sample has the same letter, then there is no statistical significance (*i.e.*, *p* > 0.05).

Furthermore, the mean percentage water contents per 100 g of honey were 17.4% ± 0% (M), 17.8% ± 0.12% (CS) and 20.4% ± 0.40% (CW). Manuka honey had high total phenol content of 103.99 ± 1.68 mg GAEs/kg compared to 86.29 ± 9.87 mg GAEs/kg for CS and 73.18 ± 8.11 mg GAEs/kg for CW. In addition a statistically significant difference (*p* < 0.05) was observed in the FRAP values (Fe^2+^ µM/kg) of 988.60 ± 0.34 (M), 1242.20 ± 0.59 (CS) and 1284.50 ± 0.28 (CW). Furthermore, strong correlations were observed between the antioxidant activity and total phenol content of each honey (Table 2). The CW honey showed the highest correlation with an R^2^ value of 0.99 followed by CS and M with R^2^ values of 0.98 and 0.86 respectively.

**Table 2:** Correlation matrix between the total phenol content of each honey and their respective anti-oxidation capacity (FRAP values).

	Correlation Coefficient		
	M	CS	CW
FRAP values (Fe^2+^ µM/kg)	0.86	0.98	0.99
Total phenol (mg GAEs/kg)	1.00	1.00	1.00

### 2.2. Antibacterial Studies

**Figure 1:**
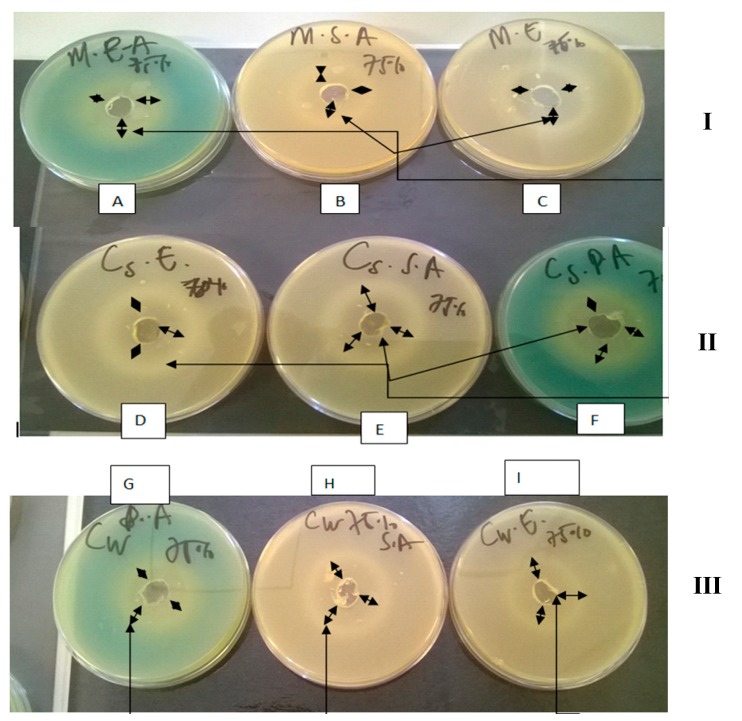
Agar plates showing the zones of inhibition generated by 75% *w*/*v* solution of the various honeys: [**I**] M honey against *Pseudomonas aeruginosa* (**A**), *Staphylococcus aureus* (**B**) and *Escherichia coli* (**C**); [**II**] CS honey against *Escherichia coli* (**D**); *Staphylococcus aureus* (**E**) and *Pseudomonas aeruginosa* (**F**); [**III**] CW honey against *Pseudomonas aeruginosa* (**G**); *Staphylococcus aureus* (**H**) and *Escherichia coli* (**I**). The long single head arrows (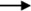) indicate the positions of the zones of inhibition whilst the short double head arrows (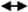) indicate the diameter of the zones of inhibition.

The three types of honey were devoid of any background bacterial contamination due to the absence of visible colonies on the agar plates after incubation. In addition, they all produced zones of inhibition (Figure 1) against the three microorganisms using the agar well diffusion [21].

For the activity of 100% pure honey, the largest zones observed were against *Escherichia coli* (1.4 × 10^9^ cfu/mL) M 35 ± 0 mm, CS 36 ± 1.0 mm and CW 36.6 ± 0.6 mm. The zones of inhibition values for *Pseudomonas aeruginosa* (1.4 × 10^9^ cfu/mL) were M 26.3 ± 0.6 mm, CS 34 ± 2.0 mm and CW 33.7 ± 3.2 mm whilst the zones of inhibition against *Staphylococcus aureus* (1.9 × 10^8^ cfu/mL) were M 18.7 ± 1.2 mm, CS 16.6 ± 0.6 mm and CW 17.0 ± 0.0 mm. The bacterial concentrations used in this study were much higher than the Clinical and Laboratory Standards Institute (CLSI) guidelines for bacterial concentration, which is recommended between 10^5^–10^6^ cfu/mL. However, this high concentration was used to simulate a highly infected, purulent chronic wound, which is always very challenging. For the diluted honey samples, zones decreased with a decrease in honey concentration (from 75% to 10%) (Table 3). Although the CW honey generally showed greater zones of inhibition, this difference was not significant. The minimum inhibitory concentrations (MICs) of CS, CW and M honey samples against *Escherichia coli*, *Staphylococcus aureus* and *Pseudomonas aeruginosa* are shown in Table 4.

All the three honey samples (CS, CW and M) at concentrations between 1.25% and 9.38% showed microbial growth of *Escherichia coli*, *Staphylococcus aureus* and *Pseudomonas aeruginosa*. However, CS, CW and M honey concentrations from 10%–75% as well as the pure honey samples showed inhibition of growth for all three bacteria investigated, indicating that the MIC for each honey against all three microorganisms was 10%.

Further, even after treatment of the various honeys (75%) with catalase (solution A), a clear zone of inhibition could be observed (Table 5a) with the highest zones observed against *Escherichia coli.* No inhibition zones were observed with the catalase/H_2_O_2_ solution (solution B) whilst very clear zones were observed when H_2_O_2_ alone (solution C), which is a known antibacterial, was used against all three organisms. However, Table 5b shows that no significant difference was observed between the 75% honey only solution and the 75% honey/catalase solution.

**Table 3:** Mean zones of inhibition (mm) of different honey concentrations against the three microbial organisms.

Concentration of Honey (% *w*/*v*)												
Bacteria	100			75			50			10		
	M	CS	CW	M	CS	CW	M	CS	CW	M	CS	CW
*E. coli*	35.0 ± 0.0	36.0 ± 1.0	36.6 ± 0.6	30.7 ± 0.6	32.7 ± 0.6	29.3 ± 0.6	26.0 ± 0.0	30.0 ± 0.0	31.0 ± 2.0	17.0 ± 1.2	17.0 ± 0.0	24.0 ± 0.0
*S. aureus*	18.7 ± 1.2	16.6 ± 0.6	17.0 ± 0.0	31.0 ± 0.0	34.0 ± 2.6	30.0 ± 0.0	28.0 ± 0.0	29.3 ± 1.5	34.0 ± 0.0	16.7 ± 0.6	15.3 ± 0.6	24.0 ± 0.0
*P. aeruginosa*	26.3 ± 0.6	34.0 ± 2.0	33.7 ± 3.2	33.0 ± 0.0	34.0 ± 1.0	37.0 ± 0.0	24.0 ± 0.6	26.0 ± 0.0	38.0 ± 0.0	10.0 ± 1.8	8.0 ± 0.0	11.0 ± 0.0

**Table 4:** Turbidity measurements of various honey solutions at different concentrations, showing the MIC values of the three types of honey against the three microorganisms [(+) indicates bacteria growth (−) indicates no growth].

	Minimum Inhibitory Concentrations of Each Honey Solution (75% *w*/*v*)																							
Bacteria	75.00			37.50			18.75			9.38			4.70				2.35					1.17		
	M	CS	CW	M	CS	CW	M	CS	CW	M	CS	CW	M	CS	CW		M		CS		CW	M	CS	CW
*E. coli*	−	−	−	−	−	−	−	−	−	+	+	+	+	+	+		+		+		+	+	+	+
*S. aureus*	−	−	−	−	−	−	−	−	−	+	+	+	+	+	+		+		+		+	+	+	+
*P. aeruginosa*	−	−	−	−	−	−	−	−	−	+	+	+	+	+	+		+		+		+	+	+	+
																					
	**Minimum Inhibitory Concentrations of Each Honey Solution (50% *w*/*v*)**																							
Bacteria	50.00			25.00			12.50			6.25			3.12				1.60							
	M	CS	CW	M	CS	CW	M	CS	CW	M	CS	CW	M	CS	CW		M		CS		CW			
*E. coli*	−	−	−	−	−	−	−	−	−	+	+	+	+	+	+		+		+		+			
*S. aureus*	−	−	−	−	−	−	−	−	−	+	+	+	+	+	+		+		+		+			
*P. aeruginosa*	−	−	−	−	−	−	−	−	−	+	+	+	+	+	+		+		+		+			
																					
	**Minimum Inhibitory Concentrations of Each Honey Solution (10% *w*/*v*)**																							
Bacteria	10.00			5.00			2.50			1.25														
	M	CS	CW	M	CS	CW	M	CS	CW	M	CS	CW												
*E. coli*	−	−	−	+	+	+	+	+	+	+	+	+												
*S. aureus*	−	−	−	+	+	+	+	+	+	+	+	+												
*P. aeruginosa*	−	−	−	+	+	+	+	+	+	+	+	+												

**Table 5:** (**a**) Zones of inhibition of various honeys after treatment with catalase; (**b**) comparison between zones of inhibition generated for 75% *w*/*v* of honey without catalase and honey with catalase, against the three bacterial organisms.

	Zones of Inhibition (mm)								
	*E. coli*			*S. aureus*			*P. aeruginosa*		
(a) Solutions	M	CS	CW	M	CS	CW	M	CS	CW
I. (2.9 mL of a 75% *w*/*v* honey solution + 0.1 mL of a 5 mg/mL catalase solution	36.0 ± 0.0	30.0 ± 0.1	30.0 ± 0.0	28.0 ± 0.2	30.0 ± 0.0	32.0 ± 0.3	27.0 ± 0.0	32.0 ± 0.3	25.0 ± 0.7
II. (2.9 mL of a 40 mM H_2_O_2_ + 0.1 mL of a 5 mg/mL catalase solution	0	0	0	0	0	0	0	0	0
III. 3mL of 40 mM H_2_O_2_ only	33.0 ± 0.0	24.0 ± 0.3	26.0 ± 0.2	29.0 ± 0.0	26.0 ± 0.7	25.0 ± 0.1	32.0 ± 0.0	25.0 ± 0.3	25.0 ± 0.6
	***E. coli***			***S. aureus***			***P. aeruginosa***		
**(b) Solutions**	**M**	**CS**	**CW**	**M**	**CS**	**CW**	**M**	**CS**	**CW**
I. (2.9 mL of a 75% *w*/*v* honey solution + 0.1 mL of a 5 mg/mL catalase solution	36.0 ± 0.0 _a_	30.0 ± 0.1 _a_	30.0 ± 0.0 _a_	28.0 ± 0.2 _a_	30.0 ± 0.0 _a_	32.0 ± 0.3 _a_	27.0 ± 0.0 _a_	32.0 ± 0.1 _a_	25.0 ± 0.7 _a_
II. 75% *w*/*v* honey solution alone	30.7 ± 0.6 _a_	32.7 ± 0.6 _a_	29.3 ± 0.0 _a_	31.0 ± 0.0 _a_	34.0 ± 2.6 _a_	30.0 ± 0.0	33.0 ± 0.0 _a_	34.0 ± 1.0 _a_	37.0 ± 0.0 _a_

The subscript “_a_” represents statistical differences calculated using the *post hoc* *t-*test (at 95% confidence interval). Since they all had the letter “_a_”, it implies there was no statistically significant (*p* > 0.05) difference between the zones generated for the 75% *w*/*v* honey solutions with catalase and 75% *w*/*v* honey solution without catalase for CS, CW and M honeys.

## 3. Discussion

Manuka honey from New Zealand has been widely studied and considered as an appropriate therapeutic standard in the treatment of ulcers, infected wounds and burns [22]. This study has demonstrated that both Cameroonian (CS and CW) honeys possess antibacterial and antioxidant activity which form part of the functional physico-chemical properties of honey considered necessary for wound healing [8]. It should be noted that the wound care benefits of honey involve other factors apart from antibacterial and antioxidation activities. The reactions of wounded tissues to interventions such as application of honey, are mediated through an array of pathophysiological pathways which is far more complex than just antibacterial and antioxidant actions of honey.

It has been reported that acceptable water contents of honey ranges between 13.66% and 25.35% [10]. Honey’s moisture content is a factor of environmental conditions as well as the harvest manipulation from beekeepers, which can vary from year to year and this makes it a critical parameter which determines the shelf life and ability to resist yeast fermentation [23]. The water contents for CS and CW honeys fell within this range, and were deemed low enough to prevent yeast fermentation and the growth of other infection causative organisms (bacteria). The high sugar content observed in both Cameroonian (CS and CW) honeys was also comparable to that of M honey, although a comprehensive qualitative sugar analysis will be required to identify the types of sugar present [24]. In addition, both CS and CW honeys were more acidic than the M honey, which will be expected to contribute to their potency against bacterial infection.

The total phenol content of 86.29 ± 9.87 (CS) and 73.18 ± 8.11 (CW) were comparable with other studies on African honeys [25]. According to literature [26,27], M honey contains a relatively high amount of phenolic compounds such as methyl syringate which possess potent superoxide scavenger activities. Alvarez-Suarez *et al.* [28] in a review article reported different profiles in M honey based on their phenolic acid contents classified into three chemotypes of *Leptospermum scoparium*. One group possessed high levels of 4-hydroxybenzoic acid, dehydrovomifoliol and benzoic acid, the second with high concentrations of kojic acid and 2-methoxybenzoic acid whilst the last group was characterized by high contents of syringic acid, 4-methoxyphenyllactic acid and methyl syringate [28].

In the current study the correlations between the total phenol content and antioxidation capacities for the CW and CS honeys were higher than M honey. This also verifies the previous study [29] in which a correlation (R^2^) of 0.87 was observed between the overall antioxidant activities of honeys and their total phenolic contents. Further, it confirms the hypothesis that both CS and CW honeys have potential antioxidation properties which may be dependent on their phenolic contents.

Although the CS and CW honeys used were not sterilized, viable counts of microorganisms were less than five colonies which could be attributed to environmental contamination. This is not surprising since the low pH, high sugar and low water contents observed do not favor bacterial growth. However, since only background bacterial contamination and not fungal contamination was investigated, it is possible that the observed colonies were due to fungal growth and therefore further investigations will be required to confirm or rule this out. The well diffusion method is reported to be better than the disc diffusion method for measuring zones of inhibition [19] and this study demonstrated the capability of Cameroonian honeys to inhibit a broad spectrum of bacterial (Gram negative and Gram positive) growth. *Escherichia coli* was observed to be most susceptible to all three (CS, CW, and M) honeys followed by *Pseudomonas aeruginosa* and *Staphylococcus aureus*. This confirms the report by Chauhan and co-workers that the most susceptible Gram-negative bacteria to honey are *Escherichia coli* and *Pseudomonas aeruginosa* [30,31]. It has been reported that the pronounced antibacterial activity of M honey directly originates from its methylglyoxal content. This non-peroxide antibacterial activity due to the presence of methylglyoxal is called the unique Manuka factor (UMF) [28]. Moreover, Kwakman and co-authors reported that neutralization of methylglyoxal totally eliminated M honey’s antimicrobial activity against *Staphylococcus aureus* but did not affect its activity against *Escherichia coli* and *Pseudomonas aeruginosa* [31]. However, the mean inhibition diameters of each honey type at different concentrations was not statistically significant (*p* > 0.05).

A consistent MIC of 10% for all three types of honey against all the three bacteria together with the zone of inhibition results, suggest that CW and CS honeys from the Adamawa region of Camerron both possess antibacterial activity similar to that of the commercially available medical grade M honey. This observation is in agreement with previously reported studies of M honey against *Pseudomonas aeruginosa* [32], and Tualang honey against *Escherischia coli*, *Staphylococcus aureus* and *Pseudomonas aeruginosa* [26] as well as M honey against *Helicobacter pylori* isolates [18]. More interestingly, the results also showed that the MIC values of 10% for both Cameroonian honeys were lower than those observed for other types of honeys from different regions of the world [7]. This suggests that the CW and CS honeys have quite potent antibacterial activity and also confirm the earlier observation that the activity of honey is a function of several factors including geographical and climatic conditions [25].

In additon, it was discovered that there was no statistically significant (*p* > 0.05) difference in the zones generated from a 75% honey solutions with and without catalase (Table 5a,b). These results suggest that both CS and CW honeys have a high non-peroxide antimicrobial activity comparable to the widely studied M honey. Since the antibacterial activity was not significantly affected by the absence of H_2_O_2_, honey’s antimicrobial effect could be attributed to various synergistically contributory factors such as phenolic compounds, pH, osmotic pressure, high sugar and low moisture contents [33]. However, apart from *Escherichia coli* which showed a slight increase in zone diameters, *Staphylococcus aureus* and *Pseudomonsa aeruginosa* showed a decrease in zone diameters, implying H_2_O_2_ is an important contributor to the antimicrobial activity of the two types of Cameroon honeys and the Manuka honey.

Generally, the processes involved in wound healing, including dressing application, should be able to deal with bacterial infection and this study has shown some modest level of this capacity for the honey samples tested. However, the events responsible for wound healing are triggered by a wide range of different ligand-receptor interactions that go beyond the antibacterial and antioxidant characteristics of the honeys tested and demonstrated in this study. Other naturally occurring materials such as amino sugar polysaccharides are reported to possess various biochemical and molecular activities that make them particularly relevant for wound healing [34]. For example the natural amino polysaccharides, chitin and chitosan, possess unique functional wound healing characteristics such as hemostasis and antibacterial activity [35]. Chitosan has been reported to be useful during the regeneration and repair of cartilage tissue regeneration and repair [36]. Chitosan also provides adequate granulation tissue formation together with angiogenesis and deposition of collagen fibers, thus allowing quicker repair of dermal and epidermal lesions [37]. Further, chitin and chitosan possess other relevant wound healing activities including polymorphonuclear cell and fibroblast activation, cytokine production, giant cell migration, acceleration of macrophage migration as well as fibroblast proliferation, granulation and vascularization [37,38].

Due to the fact that most of the observed differences in the varioius parameters investigated were not statiscally significant, it is suggested that the Cameroonian honeys possess physico-chemical and antibacterial activity similar to those of the commercially available New Zealand Manuka honey. Further, the results also suggest that geographical differences as well as honey production processes influence the antimicrobial activities of various honeys for potential wound healing to various extents. However, this will need to be confirmed with a systematic study of a wider range of African honeys for their antimicrobial activity as this study only used two samples from Cameroon in West Africa. In addition, a larger sample size would be preferable to obtain a better correlation between their physico-chemical and antimicrobial properties.

## 4. Experimental Section

### 4.1. Sample Collection and Handling

Two multi-floral 100 g honey samples were obtained from the Adamawa region of Cameroon. One of these honeys was harvested from the Martap district in Adamawa, Cameroon and was labelled Cameroon standard (CS). The other honey was purchased from an open food market also in the Adamawa region of Cameroon, and was labelled Cameroon wild (CW). A 250 g jar of Pure Gold Active 18+ Manuka Honey was obtained from Holland and Barrett (Chatham, Kent, UK). It was labelled Manuka honey (M) throughout the study. Samples were stored in their original amber containers at room temperature. All honey solutions used throughout the study were prepared in sterile distilled water and used within 2 h of preparation.

### 4.2. Physico-Chemical Properties of Honey

The density of each honey was measured relative to that of water. A graduated bottle was filled with honey to the 5 mL mark and the corresponding masses recorded using an electronic balance already calibrated with the mass of the bottle. Each recorded mass of honey was used to compute a density value using the formula: (density = mass/volume) and divided by the density of water to obtain the relative density. This was performed in triplicates for all three honeys and the mean density and standard deviation recorded for each sample.

The pH was determined according to recommendations of the Swiss Food Manual [39]. Briefly, 30% *w*/*v* honey solutions were prepared by dissolving 3 g of honey in 10 mL of sterile distilled water. The pH of each honey solution was recorded using a pH meter (Hanna Instrument pH 209, Woonsocket, RI, USA). This was performed in triplicates over a three day period and the mean and standard deviations were determined. Moisture and sugar contents were determined with reference to the International Honey Commission [39]. A Xylem Brand 44-501 Abbe’ refractometer (Bellingham and Stanley, Kent, UK) was used to determine the refractive index and the Brix 0 value of each sample in triplicates. Recorded measurements were then corrected for the standard temperature of 20 °C by adding a correction factor of 0.00023/°C. The Wedmore E.B. Bee World 36.197 1955 table was used to translate the respective refractive index values into the moisture content while the Brix 0 values were recorded as percentage sugar content.

### 4.3. Total Phenol Content

This was estimated using a modified version of the original spectrophotometric Folin-Ciocalteau method [40]. 2 g of each honey was dispersed in sterile distilled water and volume made up to 10 mL (20% *w*/*v* stock solutions). Aliquots (100 µL) of each stock solution was then mixed with 50 µL of Folin-Ciocalteau’s phenol reagent. After 3 min, 300 µL of 20% sodium carbonate (Na_2_CO_3_) solution was added to the resulting mixture with continuous shaking and incubated at room temperature for 15 min. The reaction mixture was kept in the dark for 90 min and the absorbance measured at 725 nm using a Jenway 6315 UV-Vis Spectrophotometer (Essex, UK). A standard calibration curve was generated using gallic acid at concentrations of 0, 50, 100, 150, 250 and 500 μg/mL. Total phenol content (mean ± standard deviation) was expressed as mg of gallic acid equivalents per kg of honey (mg GAEs/kg).

### 4.4. Total Anti-Oxidant Power

This was determined using ferric reducing ability of plasma (FRAP) assay [41]. This method is based on the principle that in the presence of anti-oxidants, a ferric 2,4,6-tripyridyl-s-triazine complex (Fe^3+^-TPTZ) will be reduced to its ferrous colored form (Fe^2+^-TPTZ). A working FRAP reagent was prepared by mixing 25 mL of 300 mM acetate buffer (pH 3.6) with 2.5 mL of 10 mM TPTZ solution (T1253 Sigma Aldrich, Gillingham, UK) made in a 40 mM solution of hydrochloric acid (HCl). To this solution, 2.5 mL of 20 mM ferric chloride (FeCl_3_) was added. For the test samples, 10% *w*/*v* honey stock solutions were prepared by mixing 1 g of honey into sterile distilled water and the volume made up to 10 mL. 30 µL of the 10% *w*/*v* solution was added to 1 mL of the working FRAP reagent and absorbance measured at 593 nm for time 0 min. The mixture was then incubated for 4 min at 37 °C and absorbance was again measured at 593 nm. A 1 mM aqueous ferrous sulfate (FeSO_4_·7H_2_O) solution was used to generate a calibration curve within the concentration range of 100 to 1000 µM. FRAP values were recorded and expressed as micromoles of ferrous equivalent (µM Fe[II]) per kg of honey.

### 4.5. Bacterial Growth and Maintenance

Three type cultured bacteria strains of *Staphylococcus aureus* (A29213), *Escherichia coli* (DTCC25922) and *Pseudomonas aeruginosa* (A10145) (available from the Microbiology Laboratories, University of Greenwich, London, UK), were prepared from their respective stored cultures.

Nutrient broth was initially prepared by dissolving 40 g in 1 L of water and boiled until a clear solution was obtained, bottled and autoclaved to sterilize. Next, 1 mL of each isolate was mixed with 10 mL of sterilized nutrient broth and incubated at 37 °C for 24 h. For bacterial concentration, a plate count serial dilution method was used to estimate the quantity of bacterial colony forming units (cfus) for each isolate, by doing a 10^6^ dilution [1]. Here a spread-plate technique was used for viable bacteria count, by plating 100 µL of the various dilutions onto the corresponding labelled sterile agar plates and then spreading with a sterile glass rod to ensure proper growth of the bacteria, making sure the diluted inoculums spread onto the plates were well taken by the set agar. For this step only one overnight (16 h) culture of each isolate was performed and serial dilution plate counts were always undertaken. Formed colonies visible to the naked eye were then counted and the number of cfus on the plates were determined by multiplying this count (cfu) by the total dilution factor of the solution.

### 4.6. Background Bacterial Contamination of Honey

10 g of each honey sample was dissolved in 45 mL of sterilized PBS (pH 7.1) to obtain a working stock solution (90% *w*/*v*) for each honey. This was then diluted (10 times) and 100 µL plated unto nutrient agar Petri dishes and incubated for 24 h at 37 °C. Microorganisms present in each type of honey were expressed as colony-forming units per gram (cfu/g) of honey [42].

### 4.7. Agar Well Plate Diffusion Assay Method (Zone of Inhibition)

Zones of inhibition generated by the various honeys were used as a measure of their antibacterial activity. Overnight microbial cultures in sterile Muller Hinton Agar (MHA) were prepared in separate sterile Petri dishes as previously reported [30]. 100 µL of each overnight (16 h) microbial culture was added to a separate 100 mL sterile Muller Hinton Agar (MHA), thoroughly mixed and poured into labelled sterile Petri dishes [30]. Sterile cork borers (12 mm diameter) were used to bore a single well into each agar plate. 100 µL of honey solutions (1%–75% *w*/*v*) and pure honey (0.5 g) were transferred into the 12 mm wells in the assay plates and incubated for 24 h at 37 °C and zones of inhibition measured with a ruler (*n* = 3). Sterile water was used as a control. A serial dilution plate count was then performed and incubated overnight to determine the concentration of bacteria used. Further, antibacterial activity of each pure honey was determined by dispensing 0.5 g into individual 12 mm wells as above. A serial dilution plate count was then performed and incubated overnight to determine the concentration of bacteria used.

### 4.8. Minimum Inhibitory Concentration (MIC)

The MIC was used to determine the lowest effective concentration of the three types of honey against *Staphylococcus aureus*, *Escherichia coli* and *Pseudomonas aeruginosa* using a modified broth dilution technique [43]. The previous honey concentrations (10%, 50% and 75% *w*/*v*) that showed significant antibacterial zones of inhibition were selected for determination of MIC.

Working overnight bacteria cultures were prepared and serial dilution plate counts were always performed to determine the bacteria concentrations used. Muller Hinton Broth (MHB) (2 mL) was pipetted into four sterile screw-capped test tubes (15 mL) and labelled 1 to 4 respectively. 4 mL of a 10% *w*/*v* stock solution (prepared by dissolving 1 g of honey in 10 mL MHB) was pipetted into tube 1 and a two-fold serial dilution performed for the remaining labelled pre-filled tubes by transferring 2.0, 1.0 and 0.5 mL of the 10% honey stock solution respectively and diluted to a total volume of 6 mL to yield serially diluted concentrations of 10.00%, 5.00%, 2.50% and 1.25%. 100 µL of the three microorganisms was added to each of the serial dilutions (total of 12 samples). The tubes were vortexed and incubated for 24 h at 37 °C. The same procedure was repeated for 75% (with serial dilutions of 37.50%, 18.75%, and 9.38%, 4.70%, 2.35% and 1.17%) and 50% (with serial dilutions of 25.00%, 12.50%, 6.25%, 3.12% and 1.60%) stock solutions. A tube containing only broth was used as a negative control and another containing broth and 100 µL of microbes served as a positive control. A clear tube was reported as negative (−) while a turbid cloudy solution indicated growth (+). To confirm if the MICs were bactericidal or bacteriostatic, the incubated samples were plated onto sterile nutrient agar plates and incubated overnight for confirmation of growth.

### 4.9. Non-Peroxide Antimicrobial Activity

To confirm the contribution of H_2_O_2_ or non-peroxides present in honey to the antimicrobial activity, honey solutions containing catalase and bacteria were prepared in MHB, as previously described [25]. Catalase (lyophilized powder, ≥10,000 U/mg protein, C40 Sigma-Aldrich, Gillingham, UK) was introduced to break down H_2_O_2_ present in the various honeys, and then used to generate zones of inhibition. Three tubes of test solutions were prepared and labelled as follows: tube A, (2.9 mL of a 75% *w*/*v* honey solution + 0.1 mL of a 5 mg/mL catalase solution); tube B (2.9 mL of a 40 mM H_2_O_2_ + 0.1 mL of a 5 mg/mL catalase solution) and tube C (3 mL of 40 mM H_2_O_2_ only). All three solutions were then tested in a similar manner using agar well diffusion assays on individual MHB plates and the resulting diameters of visible zones of inhibition recorded. The observed zones were compared to the previously generated zones from the initial 75% honey solutions prepared above.

### 4.10. Data Analysis

A one-way analysis of variance (ANOVA) (Excel, Microsoft Corporation, UK) was used to compare the results for the three types of honey at a significant level of *p* < 0.05. A post-hoc two tailed *t*-test was performed to determine which parameters showed significant differences. In addition, Pearson’s correlation analysis (Excel, Microsoft Corporation, Reading, UK) was performed to evaluate the association between the phenolic content of each honey and its antioxidation capacity as well as the correlation between the diameter (mm) of inhibition zones observed and the MIC.

## 5. Conclusions

This study has demonstrated that honeys from the Adamawa region of Cameroon in West Africa have similar properties to the commercially available Manuka honey. These African honeys demonstrated physico-chemical properties reported to be relevant in part to wound healing in the literature, as well as antimicrobial activities against three common wound infection causative bacteria; *Escherichia coli*, *Pseudomonas aeruginosa* and *Staphylococcus aureus*. The study also concluded that Cameroonian honeys are as effective as Manuka in reducing bacteria load with an MIC of 10% *w*/*v* and both were observed to exhibit non-peroxide antimicrobial activity.

With the current global concern about the rise of superbugs with high resistance to first line antibiotics currently used clinically, it is not surprising that there is now significant interest in natural antibacterial sources such as honey, plants and maggots that were used in ancient times. Among them, honey has made significant impact in both laboratory and clinical trials with some products already on the market. For the African subcontinent, the potential of using honey, perhaps complimentary to other antimicrobial dressings, is enormous, given the fact that it is readily available in most parts of Africa and relatively cheap, compared to mainline antibiotics. In addition, natural products such as honey are readily accepted by most indigenes from a cultural point of view, making usage in treating serious wound infections a positive prospect in the near to medium term future.

However, there may be factors in honey that are capable of both antibacterial activity and enhancing healing through components that are as yet unknown. Further extensive studies to identify these components that could kill antibiotic resistant bacteria among others, and test them clinically, are therefore required and it is hoped that this study will form part of this evolution. Future studies will seek to explore the identification of such functional components in a wide range of African honeys and to test their biological activities.
